# Supplementary material for: HIVprotI: an integrated web based platform for prediction and design of HIV proteins inhibitors
Source: J Cheminform. 2018 Mar 9;10:12. doi: 10.1186/s13321-018-0266-y (PMC5845081; doi:10.1186/s13321-018-0266-y)
Supplement: Supplementary file 2 — Additional file 2. Source code of HIVProtI web server. [file 13321_2018_266_MOESM2_ESM.zip › HIVprotI_Source-code/hivprotI/weka-3-6-6/doc/weka/core/InstanceComparator.html]

InstanceComparator


---


|  |  |  |
| --- | --- | --- |
| **Overview** \| **Package** \| **Class** \| **Tree** \| **Deprecated** \| **Index** \| **Help** \| **Weka's home** | |  |
| **PREV CLASS**   **NEXT CLASS** | **FRAMES**    **NO FRAMES**     **All Classes** |  |
| SUMMARY: NESTED \| FIELD \| CONSTR \| METHOD | DETAIL: FIELD \| CONSTR \| METHOD |  |


---


## weka.core Class InstanceComparator

```
java.lang.Object
  weka.core.InstanceComparator
```

**All Implemented Interfaces:**: java.io.Serializable, java.util.Comparator, RevisionHandler

---

``` public class InstanceComparator extends java.lang.Object implements java.util.Comparator, java.io.Serializable, RevisionHandler ```

A comparator for the Instance class. it can be used with or without the
class label. Missing values are sorted at the beginning.  
Can be used as comparator in the sorting and binary search algorithms of
`Arrays` and `Collections`.

**Version:**
:   $Revision: 1.4 $

**Author:**
:   FracPete (fracpete at cs dot waikato dot ac dot nz)

**See Also:**: `Instance`, `Arrays`, `Collections`, Serialized Form

---

**Constructor Summary**

`InstanceComparator()`
  
          initializes the comparator and includes the class in the comparison

`InstanceComparator(boolean includeClass)`
  
          initializes the comparator


| **Method Summary** | |
| --- | --- |
| `int` | `compare(java.lang.Object o1, java.lang.Object o2)`             compares the two instances, returns -1 if o1 is smaller than o2, 0 if equal and +1 if greater. |
| `boolean` | `getIncludeClass()`             returns TRUE if the class is included in the comparison |
| `java.lang.String` | `getRevision()`             Returns the revision string. |
| `static void` | `main(java.lang.String[] args)`             for testing only. |
| `void` | `setIncludeClass(boolean includeClass)`             sets whether the class should be included (= TRUE) in the comparison |

**Methods inherited from class java.lang.Object**

`equals, getClass, hashCode, notify, notifyAll, toString, wait, wait, wait`

**Methods inherited from interface java.util.Comparator**

`equals`

**Constructor Detail**

### InstanceComparator

```
public InstanceComparator()
```

:   initializes the comparator and includes the class in the comparison

---


### InstanceComparator

```
public InstanceComparator(boolean includeClass)
```

:   initializes the comparator


**Method Detail**

### setIncludeClass

```
public void setIncludeClass(boolean includeClass)
```

:   sets whether the class should be included (= TRUE) in the comparison

    :   **Parameters:**: `includeClass` - whether to include the class in the comparison

---


### getIncludeClass

```
public boolean getIncludeClass()
```

:   returns TRUE if the class is included in the comparison

---


### compare

```
public int compare(java.lang.Object o1,
                   java.lang.Object o2)
```

:   compares the two instances, returns -1 if o1 is smaller than o2, 0
    if equal and +1 if greater. The method assumes that both instance objects
    have the same attributes, they don't have to belong to the same dataset.

    :   **Specified by:**: `compare` in interface `java.util.Comparator`
    :   **Parameters:**: `o1` - the first instance to compare: `o2` - the second instance to compare **Returns:**: returns -1 if o1 is smaller than o2, 0 if equal and +1 if greater

---


### getRevision

```
public java.lang.String getRevision()
```

:   Returns the revision string.

    :   **Specified by:**: `getRevision` in interface `RevisionHandler`
    :   **Returns:**: the revision

---


### main

```
public static void main(java.lang.String[] args)
                 throws java.lang.Exception
```

:   for testing only. takes an ARFF-filename as first argument to perform
    some tests.

    :   **Throws:**: `java.lang.Exception`


---


|  |  |  |
| --- | --- | --- |
| **Overview** \| **Package** \| **Class** \| **Tree** \| **Deprecated** \| **Index** \| **Help** \| **Weka's home** | |  |
| **PREV CLASS**   **NEXT CLASS** | **FRAMES**    **NO FRAMES**     **All Classes** |  |
| SUMMARY: NESTED \| FIELD \| CONSTR \| METHOD | DETAIL: FIELD \| CONSTR \| METHOD |  |


---
